# Validation of Cardiovascular Magnetic Resonance–Derived Equation for Predicted Left Ventricular Mass Using the UK Biobank Imaging Cohort: Tool for Donor-Recipient Size Matching

**DOI:** 10.1161/CIRCHEARTFAILURE.119.006362

**Published:** 2019-06-12

**Authors:** Kenneth Fung, Caitlin Cheshire, Jackie A. Cooper, Pedro Catarino, Stefan K. Piechnik, Stefan Neubauer, Sai Bhagra, Stephen Pettit, Steffen E. Petersen

**Affiliations:** 1 William Harvey Research Institute, NIHR Barts Biomedical Research Centre, Queen Mary University of London, Charterhouse Square, United Kingdom (K.F., J.A.C., S.E.P.).; 2 Barts Heart Centre, St Bartholomew’s Hospital, Barts Health NHS Trust, London, United Kingdom (K.F., S.E.P.).; 3 Advanced Heart Failure and Transplant Unit, Royal Papworth Hospital NHS Foundation Trust, Cambridge, United Kingdom (C.C., P.C., S.B., S.P.).; 4 NIHR Oxford Biomedical Research Centre, Division of Cardiovascular Medicine, University of Oxford, United Kingdom (S.K.P., S.N.).

**Keywords:** body weight, cardiovascular diseases, heart transplantation, hospital, United Kingdom

## Abstract

**Background:**

Current guidance from International Society for Heart and Lung Transplantation recommends using body weight for donor-recipient size matching for heart transplantation. However, recent studies have shown that predicted heart mass, using body weight, height, age, and sex, may represent a better method of size matching. We aim to validate a cardiovascular magnetic resonance (CMR)–derived equation for predicted left ventricular mass (LVM) in a cohort of normal individuals in the United Kingdom.

**Methods:**

This observational study was conducted in 5065 middle-aged (44–77 years old) UK Biobank participants who underwent CMR imaging in 2014 to 2015. Individuals with cancer diagnosis in the previous 12 months or history of cardiovascular disease were excluded. Predicted LVM was calculated based on participants’ sex, height, and weight recorded at the time of imaging. Correlation analyses were performed between the predicted LVM and the LVM obtained from manual contouring of CMR cine images. The analysis included 3398 participants (age 61.5±7.5 years, 47.8% males).

**RESULTS:**

Predicted LVM was considerably higher than CMR-derived LVM (mean±SD of 138.8±28.9 g versus 86.3±20.9 g). However, there was a strong correlation between the 2 measurements (Spearman correlation coefficient 0.802, *P*<0.0001).

**Conclusions:**

Predicted LVM calculated using a CMR-derived equation that incorporates height, weight, and sex has a strong correlation with CMR LVM in large cohort of normal individuals in the United Kingdom. Our findings suggest that predicted heart mass equations may be a valid tool for donor-recipient size matching for heart transplantation in the United Kingdom.

## Abstract 2

Supplemental Digital Content is available in the text.

**WHAT IS NEW?**

- In this large cohort study, we demonstrated that predicted left ventricular (LV) mass using sex, height, and weight has strong correlation with LV mass measured by cardiovascular magnetic resonance (CMR) imaging.
- The correlation of CMR-measured LV mass with predicted LV mass is stronger compared with that of weight or height.
- There is poor absolute numerical agreement between predicted LV mass and CMR-measured LV mass (absolute difference in mean values=62 g for men, 44 g for women).

**WHAT ARE THE CLINICAL IMPLICATIONS?**

- Our findings challenge the clinical practice of using weight or height alone for donor-recipient size matching in heart transplantation.
- Predicted heart mass using CMR-derived equations seems to offer a better technique for donor-recipient size matching than using height or weight alone.
- The equations for predicted heart mass, which were derived from a multiethnic group, are translatable to a predominately white population.

**See Editorial by Tremblay-Gravel and Khush**

Size matching is an important consideration in heart transplantation. At a simplistic level, a recipient should receive a donor heart that is large enough to generate adequate cardiac output and overcome recipient pulmonary vascular resistance but small enough to fit into the mediastinal space and allow the chest to be closed. Current guidelines for donor-recipient size matching are based on body weight alone. The International Society for Heart and Lung Transplantation^1^ recommends (1) that it is safe to use hearts from donors whose body weight is <30% below that of the recipient, (2) that a heart from a male donor of average body weight (70 kg) is acceptable for all recipients, and (3) that in cases where the donor is female, then her body weight should not be >20% lower than a male recipient. These recommendations are based on expert consensus opinion and small observational studies from the early 1990s.^2,3^

It is recognized that body weight is not a good marker of heart mass. This is a particular concern with the rising prevalence of obesity in the 21st century.^4,5^ Ventricular mass varies significantly with age and sex.^6–8^ Equations to predict left and right ventricular heart mass (predicted heart mass [PHM]) have been derived from regression models of cardiovascular magnetic resonance (CMR) imaging using a large subset of participants in the American, population-based MESA (Multi-Ethnic Study of Atherosclerosis) cohort.^9,10^ These equations incorporate height, weight, age and sex to determine PHM. Total PHM has been proposed as an alternate method to evaluate donor-recipient size matching for heart transplantation.

Total PHM as a tool for size matching in heart transplantation has been explored in the United Network for Organ Sharing dataset. There was no association between post-transplant survival and undersizing by body weight difference, but there was lower post-transplant survival with undersizing by PHM difference. PHM difference appeared to modulate the lower post-transplant survival associated with female donor into male recipient sex mismatching.^11,12^ In addition, undersizing by PHM difference seems to be associated with higher rates of severe primary graft dysfunction after heart transplantation.^13^

Although PHM equations are a promising tool for size matching in heart transplantation, PHM equations were not derived for this purpose, and there is no data to support the validity of this approach outside the United States.^11^ In addition, PHM equations were derived from a single study based in the United States and have not been externally validated. This study aims to validate a CMR-derived equation for predicted left ventricular mass (LVM) in a large population of normal individuals within the UK Biobank imaging study.

## Methods

### Data Access

The data used in this study are available to all bona fide researchers via application to the UK Biobank in accordance with their approval criteria. Information for the detailed access procedure can be found at https://www.ukbiobank.ac.uk/using-the-resource/.

### Study Participants

The first 5065 participants from the UK Biobank imaging substudy (https://imaging.ukbiobank.ac.uk) enrolled between 2014 and 2015 were included. These participants were initially part of the 500 000+ men and women aged 40 to 69 years recruited in 2006 to 2010 into the UK Biobank study, which is a large prospective study with a wealth of physical, genetic, and clinical data for health-related research. The imaging substudy aims to assess over 100 000 participants for a range of imaging, including CMR, by 2023.^14^ The UK Biobank study was approved by the North West Multi-Centre Research Ethics Committee and written informed consent was obtained from all individuals at recruitment to participate in the UK Biobank study. All methods were performed in accordance with the relevant guidelines and regulations.

### Exclusion Criteria

Participants with a history of angina, myocardial infarction, stroke, and atrial fibrillation were excluded. We also excluded those with a reported diagnosis of cancer in the previous 12 months before CMR imaging. These data were collected based on self-reported questionnaires and nurse-led verbal interviews on past and current medical conditions. In addition, diagnoses were captured from Hospital Episode Statistics records according to the *International Classification of Diseases, Ninth and Tenth Revisions*.^15^ We also utilized the algorithmic definitions for myocardial infarction^16^ and stroke^17^ developed by UK Biobank that incorporates data from hospital admissions and death registries. No one declared to be pregnant at the time of imaging. Finally, individuals who weighed >135 kg were excluded.

### CMR Acquisition and Derived Parameters

The UK Biobank CMR imaging protocol and image analysis have been described in detail previously.^18,19^ Briefly, CMR examinations were performed with a 1.5 Tesla scanner (MAGNETOM Aera, Syngo Platform VD13A; Siemens Healthcare, Erlangen, Germany) in a single dedicated center for UK Biobank imaging in Cheadle, United Kingdom. The manual analyses of CMR images were previously performed using cvi42 postprocessing software (Version 5.1.1, Circle Cardiovascular Imaging, Inc, Calgary, Canada) between 8 readers located in 2 core laboratories based on standard operating procedures for analysis. The end-diastolic frame was nominally defined as the first frame of the image series. Papillary muscles were included as blood pool and excluded from mass. The derived left ventricular (LV) parameters included: end-diastolic volume, end-systolic volume, stroke volume, mass and ejection fraction. A healthy subcohort was previously used to derive normal reference ranges^19^ and provided cutoffs for our study whereby individuals with LV parameter values outside their sex-specific reference ranges were removed.

### Predicted LVM

Predicted LVM was calculated using the equation developed from MESA^9^: a×(height in meters)^0.54^×(weight in kilograms)^0.61^, where a=6.82 for women and 8.25 for men. Physical measurements were recorded on the day of imaging with height being measured to the nearest centimeter in a barefoot standing position using a Seca 202 device (Seca, Birmingham, United Kingdom). Body weight was measured, to the nearest 0.1 of a kilogram, using a Tanita BC-418MA body composition analyzer. Participants were asked to remove their shoes and heavy outer clothing before weight measurements were taken.

### Statistical Analyses

LVM was assessed for normality using histograms and quantile-quantile plots. Continuous variables were summarized as mean±SD or median (interquartile range), whereas categorical data were displayed as absolute counts (percentages). Linear regression analysis was performed to compare predicted LVM and CMR LVM. Bland-Altman plot using log-transformed predicted LVM and log-transformed CMR LVM were used to assess for bias and limits of agreement. Log-transformation was performed to adjust for the relative difference that exists between the derived LVM in MESA and UK Biobank. R (version 3.5.1) software^20^ was used for all statistical analyses, and *P*<0.05 was considered statistically significant.

## Results

### Baseline Demographics

**Figure 1.**
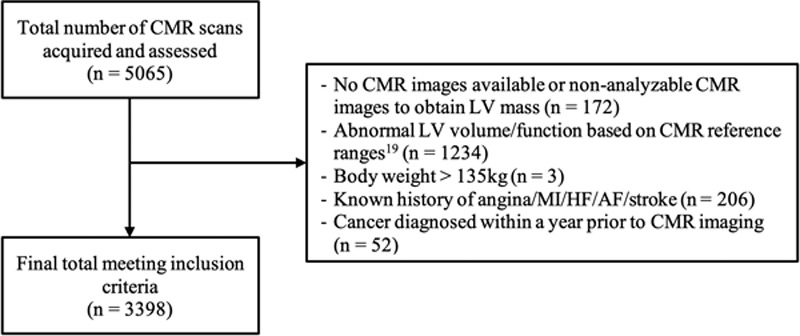
**UK Biobank participant selection flowchart.** AF indicates atrial fibrillation; CMR, cardiovascular magnetic resonance; HF, heart failure; LV, left ventricular; and MI, myocardial infarction.

Of the 5065 participants, we initially excluded those with either no available images or nonanalyzable images (n=172). We excluded individuals with LV parameters that lie outside the normal ranges (n=1234). We excluded 3 individuals who weighed >135 kg. A further 258 participants were excluded due to a history of cardiovascular disease or a diagnosis of cancer in the preceding 12 months. After these exclusions, a total of 3398 individuals (age 61.5±7.5 years, 47.8% males) were included in our study (Figure 1), and their characteristics are summarized in the Table. The median (interquartile range) body mass index was 25.3 (5.0) kg/m^2^. The mean LVM obtained from the manual analysis of CMR images was 102.9±16.4 g for men and 71.1±10.4 g for women, whereas the mean predicted LVM in the same cohort was 165.0±16.3 g for men and 114.8±12.3 g for women.

**Table.**
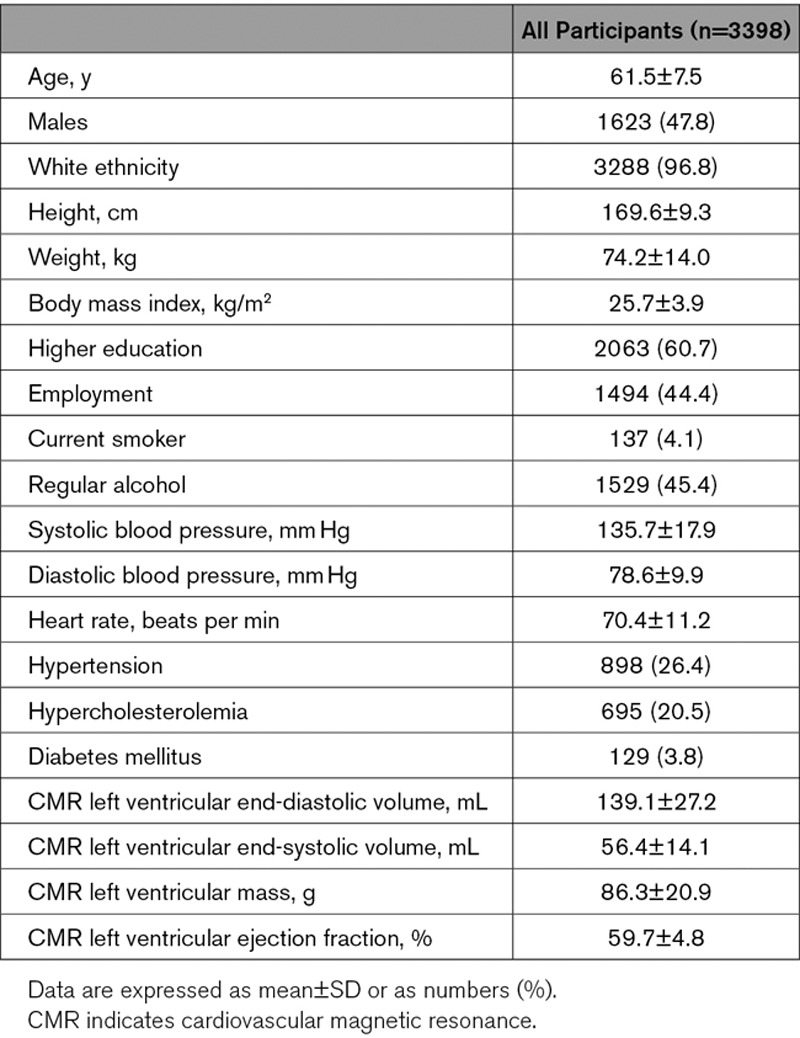
UK Biobank Participant Characteristics

### Correlation Between Predicted LVM and CMR LVM

**Figure 2.**
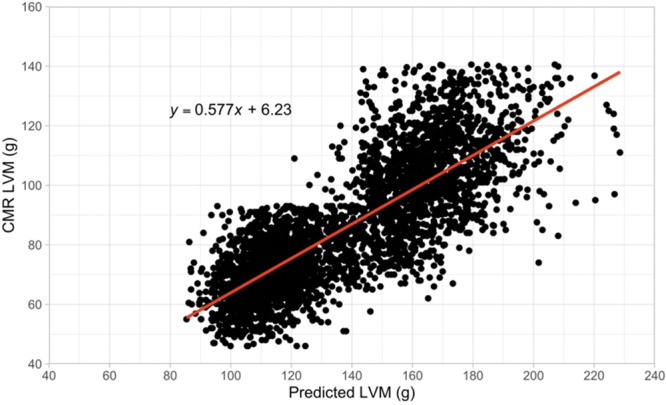
**Correlation of predicted left ventricular mass (LVM) and cardiovascular magnetic resonance (CMR) LVM.** A strong correlation between the predicted LVM calculated using a CMR-derived equation and CMR-measured LVM was found with a Spearman’s correlation coefficient 0.802 (*P*<0.0001).

The predicted LVM calculated using the equation has strong positive correlation with the measured LVM from CMR images in our cohort (Figure 2). The Spearman correlation coefficient was 0.802 (*P*<0.0001) between predicted LVM and CMR LVM. The sex-specific correlation for men and women between these 2 masses was more modest with coefficients of 0.431 (*P*<0.0001) and 0.401 (*P*<0.0001), respectively (Figure I in the Data Supplement).

**Figure 3.**
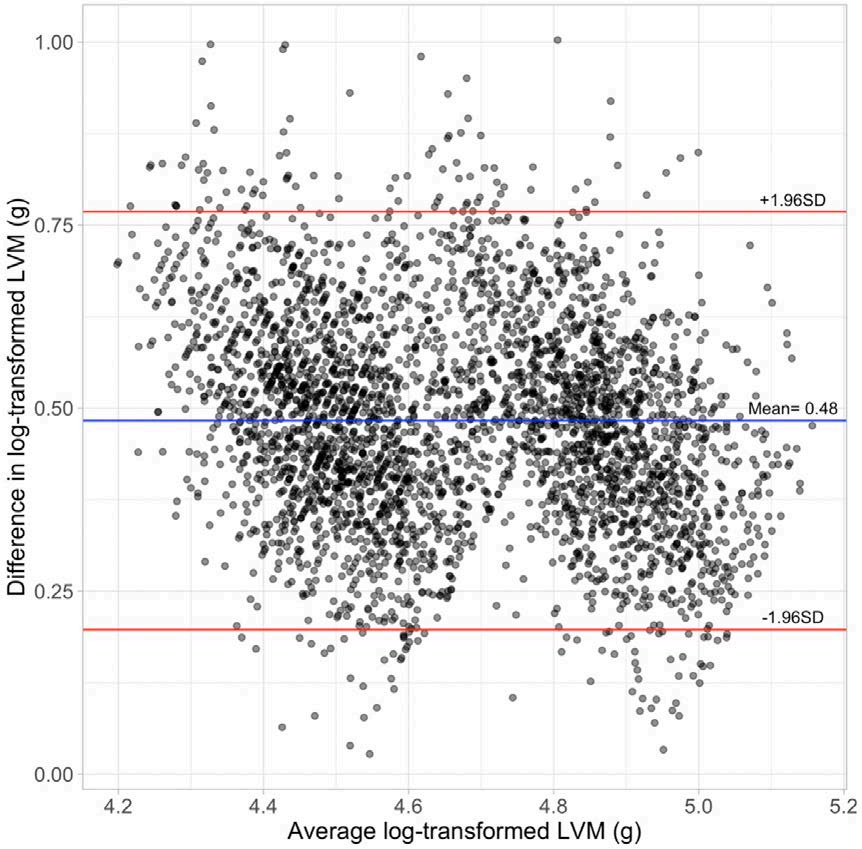
**Bland-Altman plot for the differences between log-transformed predicted left ventricular mass (LVM) and cardiovascular magnetic resonance (CMR) LVM.** Blue line represents the mean of the differences between log-transformed predicted LVM and CMR LVM. Red lines denote the upper and lower limits of agreement (mean difference±[1.96×SD]), where SD is the SD of the differences.

Bland-Altman analysis showed generally excellent agreement between the observed with predicted LVM with mean log-transformed difference of 0.48±0.15 g (Figure 3). The 95% limits of agreement between the 2 log-transformed measurements were between 0.19 g and 0.77 g. The 95% CI of the lower and upper limits of agreement were 0.186 g to 0.203 g and 0.757 g to 0.774 g, respectively.

### Correlation Between Height/Weight and CMR LVM

**Figure 4.**
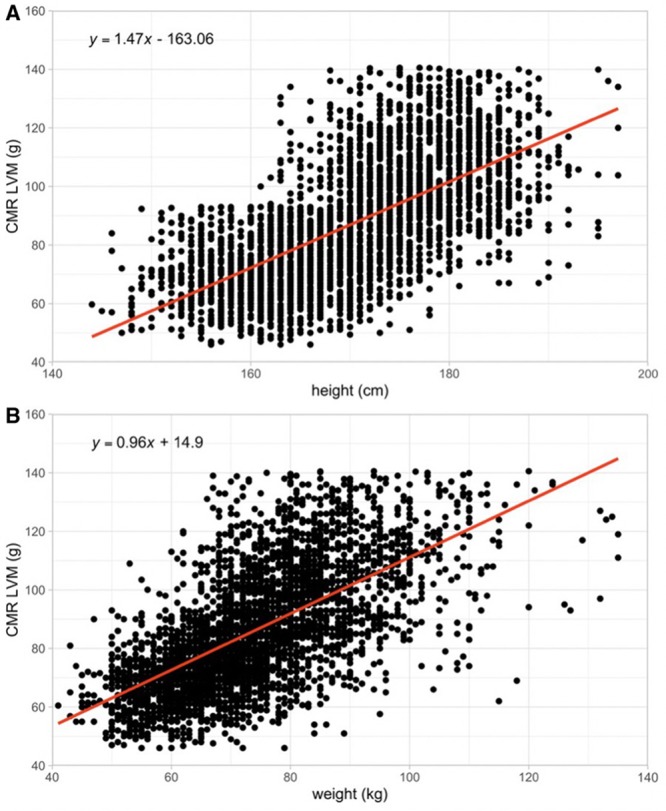
**Correlation of cardiovascular magnetic resonance (CMR) left ventricular mass (LVM) with height and weight.** Strong correlations were found for (**A**) height and (**B**) weight (Spearman correlation coefficient 0.684 and 0.665 [*P*<0.0001 for both], respectively), but the magnitude of these correlations is less compared with the correlation between predicted LVM and CMR LVM.

Given that height or weight alone is used in current clinical practice for donor-recipient size matching in heart transplantation, we, therefore, also report the correlation of these measures with CMR LVM. In our studied population, both height and weight were found to have strong positive correlations with CMR-measured LVM (Figure 4). The Spearman correlation coefficients for these measures with CMR LVM were 0.684 (*P*<0.0001) for height and 0.665 (*P*<0.0001) for weight, respectively. However, the correlations are greatly reduced when we derived for males and females. For males, the Spearman correlation coefficients were 0.235 (*P*<0.0001) for height and 0.427 (*P*<0.0001) for weight (Figures II and III in the Data Supplement). For females, the Spearman correlation coefficient was 0.199 (*P*<0.0001) for height and 0.389 (*P*<0.0001) for weight (Figures II and III in the Data Supplement).

## Discussion

We have validated an equation for prediction of LV mass in 3398 participants of the UK Biobank study. We have demonstrated that predicted LVM has a strong correlation with CMR-measured LVM, performed by manual contouring of short-axis cine images, in our study population. This finding is encouraging with regard to the use of PHM equations for donor-recipient size matching in heart transplantation.

There was a significant difference between the absolute values of predicted LVM and CMR-measured LVM (absolute difference in mean values=62 g for men, 44 g for women). This is an expected finding due to differences in the imaging protocols and analysis technique between the MESA and UK Biobank studies. The mean LVM in the MESA cohort that was used to derive the PHM equations, but without traditional cardiovascular risk factors, was 163.8±35.8g for men and 113.6±24.2 g for women.^7^ However, the mean LVM in the UK Biobank healthy subset was lower (103±21 g for men, 70±13 g for women), and therefore, much of the absolute difference may represent differences in imaging and analysis techniques. Calculated LVM has been previously shown to be greater when measured from traditional gradient-echo images, which was used in the MESA study, compared with images acquired using balanced steady-state free precession imaging sequence.^21^

Although the age and sex ratio are comparable between the MESA and UK Biobank cohorts, there are several baseline characteristics that would also account for the higher LVM seen in the MESA cohort. First, the MESA cohort was ethnically diverse, with 39% white, 26% Hispanic, 26% black, and 13% Chinese-American participants. In contrast, 96.8% of participants in the UK Biobank study were white, and this may have contributed to lower measured LV mass. Second, in our studied populations, the MESA participants had higher body mass index (mean 28±5 versus 26±4 kg/m^2^), and third, the proportion of current smokers was 12% in MESA cohort compared with just 4% among UK Biobank participants.

### Clinical Implications

It would seem that PHM equations are well correlated with a “real-world” measurement of heart mass in a large population of normal individuals, the type of individuals who might become organ donors in unfortunate circumstances. It is important to note that the absolute value of PHM is less important than the correlation between PHM and a “real-world” measurement of heart mass. When using an equation to predict both donor and recipient matching, one is trying to judge the relative difference between donor and recipient heart mass rather than the absolute heart mass.

PHM equations may become a useful tool for size matching in heart transplantation. All required variables are available when decisions about donor-recipient suitability are being made. PHM is relatively straightforward to calculate. In addition, it would be amenable to automate calculations using an online or mobile app, and this could be incorporated with a decision support tool for healthcare professionals. Such a tool might help clinicians avoid undersizing and therefore reduce the risk of primary graft dysfunction and post-transplant mortality in the recipient. In particular, the equations may help clinicians to identify the hidden undersizing that is associated with sex mismatching of female donor hearts into male recipients.

Further work is required to determine whether size matching by PHM is the optimal metric for donor-recipient size matching in heart transplantation and whether a cutoff exists for an acceptable level of undersizing. This may require complex statistical analysis of other factors that may interact with undersizing, such as ischemic time, donor age, and recipient pulmonary vascular resistance.

### Strength and Limitations

The main strengths of this study lie in the fact that it is performed in a large cohort, and CMR data has been collected and analyzed using defined protocols to produce highly accurate and reproducible data. However, we do acknowledge some limitations in our study. The UK Biobank participants are predominately middle-aged whites, and so the findings may not be applicable to other age and ethnic groups. Cancer diagnoses and the year (or age) of diagnosis were self-reported. Therefore, we may not have captured all cancer diagnoses within 12 months of CMR imaging. Finally, we could only validate predicted LVM as right ventricular mass was not manually derived from the UK Biobank CMR images.

## Conclusions

In a large middle-aged white cohort, we have demonstrated a strong correlation between LVM predicted by a CMR-derived equation and LVM measured by CMR in a large population of normal individuals in the United Kingdom. Our findings support ongoing study of PHM equations as a tool for donor-recipient size matching in heart transplantation.

## Acknowledgments

This research has been conducted using the UK Biobank Resource under Application 2964. We thank all UK Biobank participants and staff. We also thank Nay Aung, Jose M. Paiva, Elena Lukaschuk, Mihir M. Sanghvi, Mohammed Y. Khanji, Filip Zemrak, Valentina Carapella, and Young Jin Kim for contributing significantly to the manual analysis of the UK Biobank cases.

## Sources of Funding

K. Fung is supported by The Medical College of Saint Bartholomew’s Hospital Trust, an independent registered charity that promotes and advances medical and dental education and research at Barts and The London School of Medicine and Dentistry. Drs Petersen, Piechnik, and Neubauer acknowledge the British Heart Foundation for funding the manual analysis to create a cardiovascular magnetic resonance (CMR) imaging reference standard for the UK Biobank imaging resource in 5000 CMR scans (PG/14/89/31194). The UK Biobank was established by the Wellcome Trust medical charity, Medical Research Council, Department of Health, Scottish Government, and the Northwest Regional Development Agency. It has also received funding from the Welsh Assembly Government and the British Heart Foundation.

## Disclosures

Dr Petersen provides consultancy to Circle Cardiovascular Imaging Inc, Calgary, Canada. The other authors report no conflicts.

## Supplementary Material
