# Supplementary material for: Validation of Cardiovascular Magnetic Resonance–Derived Equation for Predicted Left Ventricular Mass Using the UK Biobank Imaging Cohort: Tool for Donor-Recipient Size Matching
Source: Circ Heart Fail. 2019 Jun 12;12(12):e006362. doi: 10.1161/CIRCHEARTFAILURE.119.006362 (PMC6922072; doi:10.1161/CIRCHEARTFAILURE.119.006362)

SUPPLEMENTARY MATERIAL

Supplementary Figure 1. Correlation of predicted LVM and CMR LVM in (A) men and (B) women. Rho is the Spearman's correlation coefficient. LVM = left ventricular mass; CMR = cardiovascular magnetic resonance.

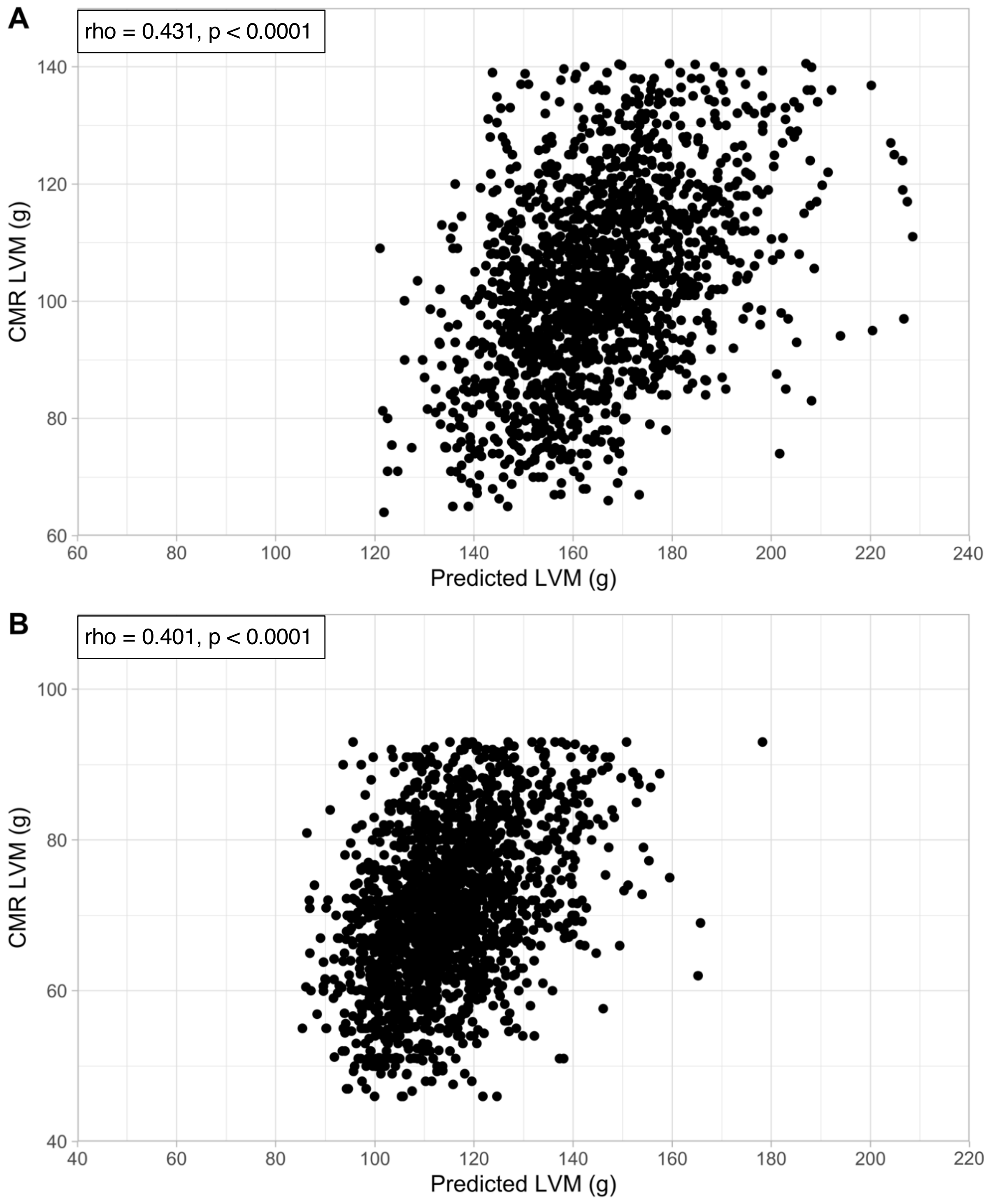

Supplementary Figure 2. Correlation of CMR LVM with weight in (A) men and (B) women. Rho is the Spearman's correlation coefficient. LVM = left ventricular mass; CMR = cardiovascular magnetic resonance.

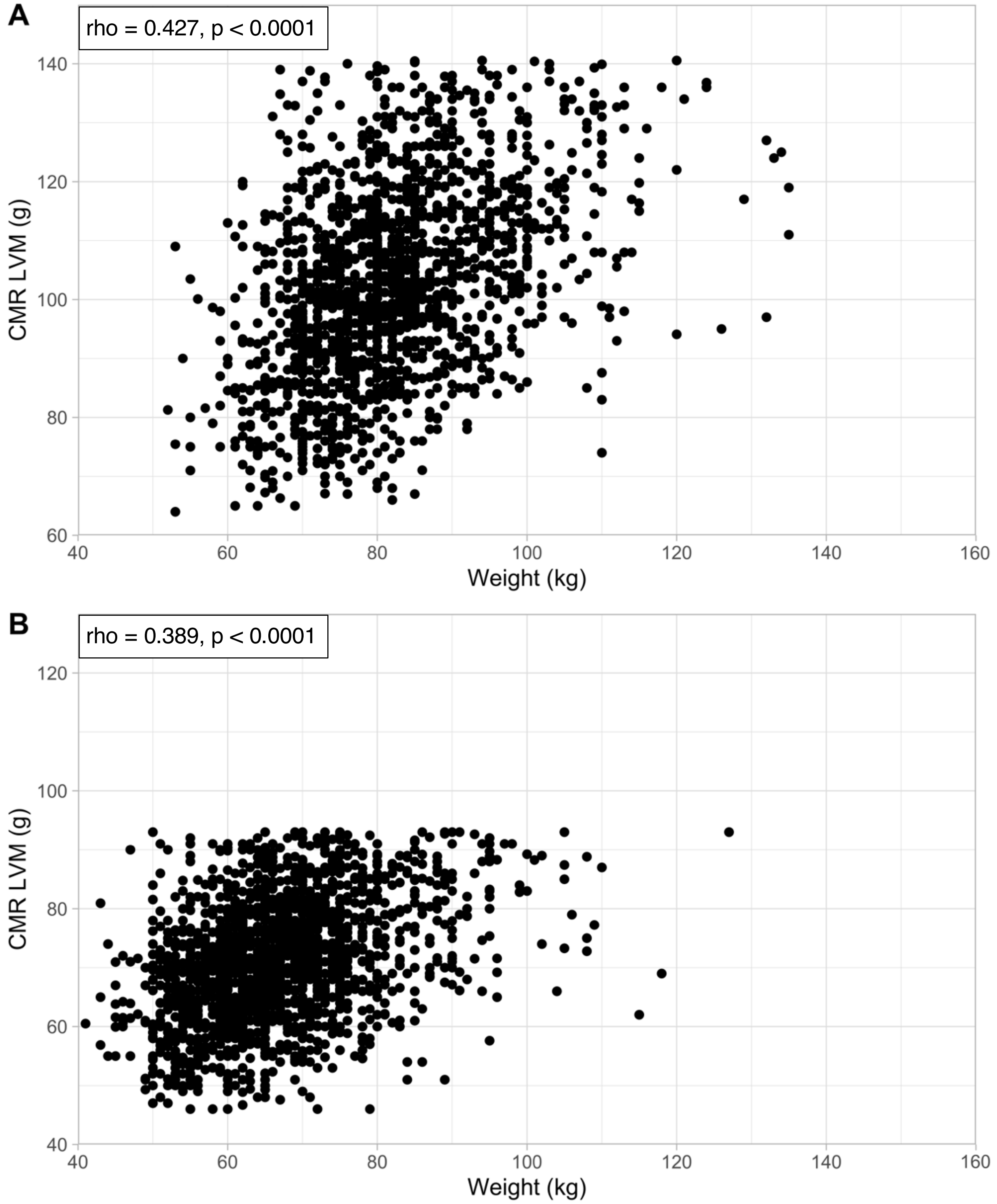

Supplementary Figure 3. Correlation of CMR LVM with height in (A) men and (B) women. Rho is the Spearman's correlation coefficient. LVM = left ventricular mass; CMR = cardiovascular magnetic resonance.

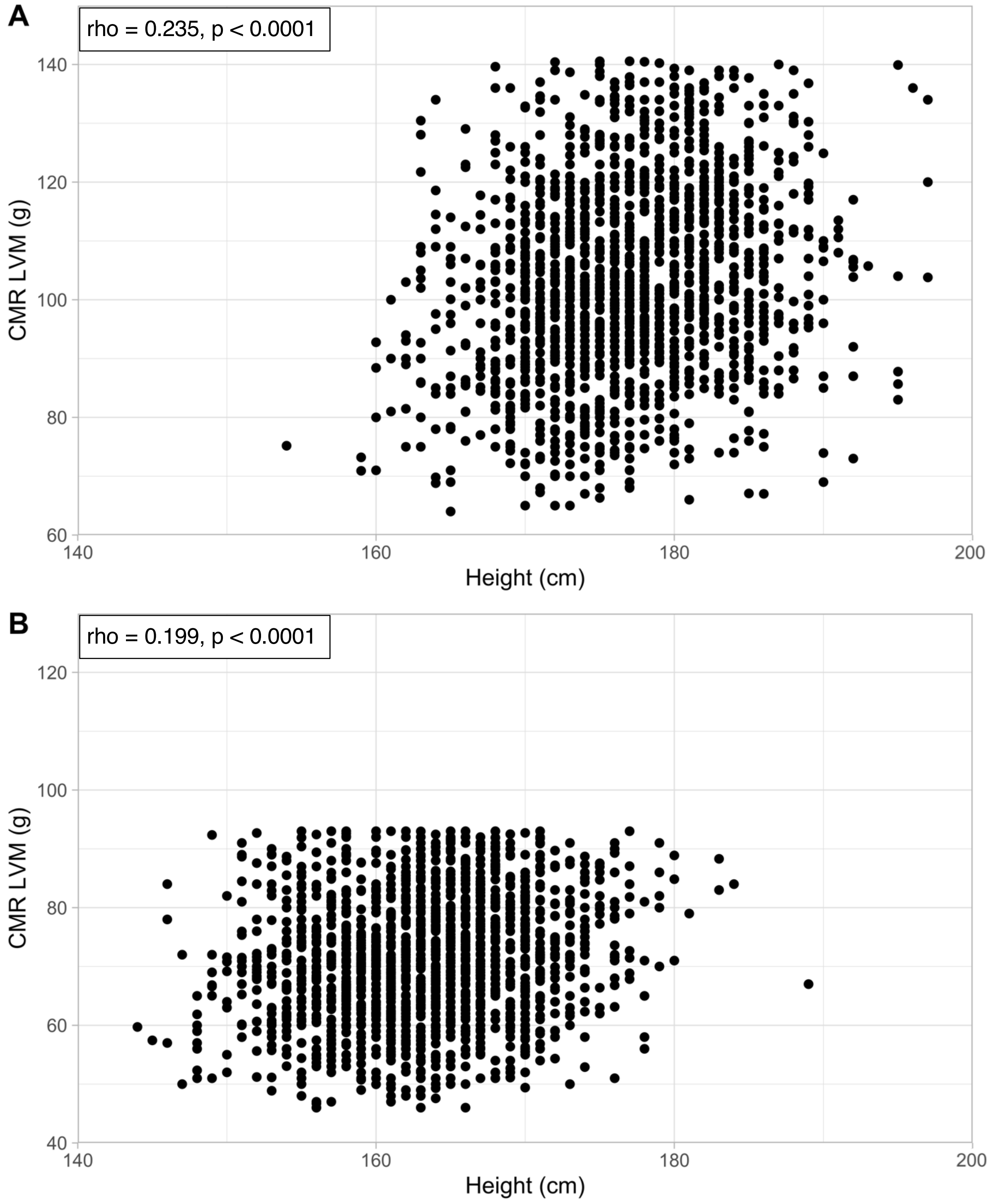

Supplement: Supplementary file 1 [file hhf-12-e006362-s001.pdf]
